# Construction of ceRNA and m6A-related lncRNA networks associated with anti-inflammation of AdipoAI

**DOI:** 10.3389/fimmu.2022.1051654

**Published:** 2023-01-10

**Authors:** Hongwen Yu, Hongle Wu, Qiuyan Xie, Zining Liu, Zehao Chen, Qisheng Tu, Jake Chen, Fuchun Fang, Wei Qiu

**Affiliations:** ^1^ Department of Stomatology, Nanfang Hospital, Southern Medical University, Guangzhou, China; ^2^ Department of Endodontics, Stomatological Hospital, Southern Medical University, Guangzhou, China; ^3^ Division of Oral Biology, Tufts University School of Dental Medicine, Boston, MA, United States

**Keywords:** AdipoAI, m6A related-lncRNA, ceRNA, lipopolysaccharide, anti-inflammation, adiponectin

## Abstract

**Background:**

Adiponectin (APN) is an endogenous adipokine secreted from adipocytes that exerts anti-inflammatory properties. AdipoAI is an orally active adiponectin receptor agonist identified by our group that can emulate APN's anti-inflammatory properties through mechanisms that are not fully understood. LncRNAs, a type of noncoding RNA more than 200 bp in length, have been demonstrated to have abundant biological functions, including in anti-inflammatory responses.

**Materials and Result:**

In the current study, we performed a lncRNA microarray in LPS-induced Raw264.7 cells that were prestimulated with AdipoAI and screened 110 DElncRNAs and 190 DEmRNAs. Enrichment analyses were conducted on total mRNAs and DEmRNAs, including GSVA, ssGSEA, GO/KEGG, GSEA, and PPI analysis. Among all these processes, endocytosis was significantly enriched. A coexpression analysis was built based on DElncRNAs and DEmRNAs. Then, using TargetScan and miRwalk to predict related microRNAs of DElncRNAs and DEmRNAs, respectively, we established competing endogenous RNA (ceRNA) networks including 54 mRNAs from 8 GO items. Furthermore, 33 m6A methylation-related marker genes were obtained from a previous study and used for the construction of an m6A-related lncRNA network by coexpression analysis. We identified FTO as the hub gene of the network and 14 lncRNAs that interacted with it. The expression levels of 10 lncRNAs selected from ceRNA and FTO-related lncRNA networks were validated with qRT‒PCR. Finally, macrophage phenotype scores showed that AdipoAI could attenuate the M2b and M2c polarization of macrophages and correlate with the above lncRNAs.

**Conclusion:**

Our work reveals that lncRNAs might be involved in the anti-inflammation process of AdipoAI in LPS-induced macrophages through the ceRNA network and the epigenetic regulation of m6A. Mechanistically, these lncRNAs associated with AdipoAI might be related to endocytosis and polarization in macrophages and provide new candidates for the anti-inflammatory application of APN and its receptor agonist.

## 1 Introduction

Inflammation is an adaptive response triggered by noxious substances and tipped balances, such as infections and injuries (1). Thus, it contributes to two-phase responses, including defending against invading pathogens and provoking pathological processes (2). LPS, a potent inducer of inflammation, contributes to the occurrence of the inflammatory response by activating macrophages and inducing a series of substances, including proinflammatory cytokines and vasoactive mediators (3). Emerging evidence has emphasized that uncontrolled production of proinflammatory cytokines may affect homeostasis and lead to serious consequences, such as septic shock (4). That is, precise tuning of pro- and anti-inflammatory mediators is pivotal for inner homeostasis maintenance.

Adiponectin (APN), an endogenous secretory protein produced by white adipose tissue (5), stimulates downstream signals and is involved in various physical functions, such as improving energy utilization and insulin sensitivity (6). Additionally, APN has been reported to have profound anti-inflammatory effects in numerous diseases, such as type 2 diabetes and periodontitis (7). Specifically, studies indicate that macrophages are the primary target of APN in the anti-inflammation process since it can modulate macrophage functions and inhibit the activation of TLR4 (8–10). However, the requirement of a high dosage of intravenous injection for a constant period as well as the technology scarcity to produce APN proteins on a large scale and with sufficiently high quality act as obstacles for putting APN into clinical practice (11). To address this scarcity, AdipoRon, the first adiponectin receptor agonist, was designed by the University of Tokyo and serves as an orally active small molecule with antidiabetic properties (12). Inspired by it, our previous study also designed a potent adiponectin receptor agonist named AdipoAI, which was proven to inhibit the LPS-induced inflammatory response in macrophages by moderating the interaction between APPL1 and the MyD88 protein (13). As a structural analogue of AdipoRon, AdipoAI showed similar characteristics of orally active acetamide small molecule compound. Interestingly, we found that AdipoAI exerted an approximately eightfold greater than AdipoRon to inhibit the gene expression of inflammatory cytokines in macrophages.

Recently, as sequencing technology has continued to evolve, an upsurge of research on noncoding RNA has appeared in various fields. LncRNAs are collectively classified as noncoding RNAs >200 nucleotides in length (14) and have been shown to play essential roles in multiple physiological activities and pathological processes, including cell differentiation, tissue organ development, and cancer metastasis (15–19). Moreover, numerous reports have shown that lncRNAs involved in such signaling pathways, including NF-kB, MAPK or TLR-related pathways, participate in regulating the inflammatory response of macrophages (20). Attention should be given to competing endogenous RNA (ceRNA) and N6-methyladenosine (m6A) methylation owing to the ceRNA network uncovering the links between mRNAs and ncRNAs (21), while m6A RNA modification serves as a major regulator for RNAs, elucidating the relationship between m6A-related RNA and others (22). Overall, ceRNA and m6A networks are regarded as an important step in discovering the molecular mechanism of lncRNAs in inflammatory homeostasis. Thus, exploring the role of lncRNAs in inflammatory homeostasis could provide novel mechanistic ideas regarding their cointeractions with other molecules.

In the current study, we aimed to further investigate whether lncRNAs contribute to the anti-inflammatory effect of AdipoAI or APN in LPS-induced macrophages. Therefore, we conducted a lncRNA microarray in LPS-induced Raw264.7 cells pretreated with AdipoAI and performed a series of bioinformatics analyses to explore the potential signaling pathways and underlying mechanisms involved in ceRNA- and m6A-related lncRNA networks. Finally, we identified and validated ten ceRNA- and m6A-related lncRNAs, which may provide new therapeutic targets for the anti-inflammatory effects of APN and its receptor agonist.

## 2 Materials and methods

### 2.1 LncRNA microarray resources and process

Raw264.7 cells were stimulated with AdipoAI for 24 h followed by incubation with 100 ng/ml LPS (E. coli 0111: B4, Sigma‒Aldrich, St. Louis, MO, USA) for an additional 6 h. Detailed information of the four groups is shown in the figure legends. Total RNA was extracted from cells, and 5 μg of total RNA from each sample was sent to Arraystar Inc. (Rockville, MD, USA) for microarray analysis. Image processing and data extraction and analysis were also performed by Arraystar Inc. Using their established protocols. Total data from 12 samples were divided into four groups: DMSO, LPS, AdipoAI and LPS+AdipoAI. Then, raw signal intensities were normalized using the quantile method provided by GeneSpring GX v12.1, while low-intensity mRNAs and lncRNAs were filtered. Multiple probes corresponding to the same gene were screened and selected randomly to remove duplications. Then, box plots were constructed by factoextra, and the FactoMineR R package was applied for principal component analysis (PCA) to assess data quality.

### 2.2 Gene set variation analysis and macrophage phenotype ssGSEA

Gene set variation analysis (GSVA) is a method that estimates the variation in pathway activity over a sample population in an unsupervised manner (23). Enriched pathways of total mRNAs extracted from 12 samples were analyzed by the GSVA R package, while “c2.cp.v7.5.1.symbols.gmt” was used as the molecular signatures’ dataset. Subsequently, GSVA scores were displayed by heatmap using the pheatmap R package, and a score matrix was formed. According to the matrix and the threshold of P value<0.05 and |log2 FC (fold change) |≥1.5, the R package limma was used to select differentially expressed pathways among three pairs of samples: DMSO vs. LPS, LPS vs. LPS+AdipoAI, and DMSO vs. AdipoAI. The EnhancedVolcano R package was used for visualization. Furthermore, common pathways among the 3 pairs were identified through a Venn plot created by the EnhancedVolcano R package, and the GSVA scores of each common pathway were shown by a heatmap generated using the pheatmap R package.

To discover the role of AdipoAI in the regulation of macrophage polarization, a gene set, including a series of macrophage phenotype markers, was obtained from a previous study (24) and is displayed in **Table 1**. After that, the GSVA R package was applied to the gene set for single-sample gene set enrichment analysis (ssGSEA) and normalization, and the scores were revealed by heatmap using the pheatmap R package.

**Table 1:** Macrophage phenotype-related genes.

Macrophage Type	M1	M2a	M2b	M2c	M2d (TAM)
**Related Genes**	TNF	IL-10	IL-10	IL-10	IL-10
	IL-1	TGFB1	IL1B	TGFB1	VEGF
	IL-6	CD206	IL-6	CD163	CD206
	IL-12	CD36	TNF	TLR1	CD204
	IL-23	IL1Ra	CD86	TLR8	CD163
	CD80	CD163	CIITA2	ARG1	ARG1
	CD86	ARG1	ARG1	GS	IDO
	CIITA	CARKL	CARKL	STAT3	STAT1
	MHC-II	STAT6	STAT3	STAT6	IRF3
	iNOS	GATA3	IRF4	IRF4	NFKB1
	PFKFB3	SOCS1	NFKB1	NFKB1	
	PKM2	PPARG			
	ACOD1				
	NFKB1				
	STAT1				
	STAT3				
	IRF-4				
	HIF1A				
	AP1				

### 2.3 Differential expression analysis

Limma is an R software package that provides differential expression analysis for microarray and high-throughput PCR data (25). DEmRNAs and DElncRNAs with a P value<0.05 and |log2 FC (fold change) |≥1.5 as thresholds were selected by the limma package from three pairs of RNA data from total samples: DMSO vs. LPS, LPS vs. LPS+AdipoAI, and DMSO vs. AdipoAI. Furthermore, specific groups of DEmRNAs and DElncRNAs were screened out for investigation of AdipoAI-related potential pathways: upregulated in DMSO vs. LPS while downregulated in both LPS vs. LPS+AdipoAI and DMSO vs. AdipoAI; and downregulated in DMSO vs. LPS while upregulated in both LPS vs. LPS+AdipoAI and DMSO vs. AdipoAI. For visualization, the EnhancedVolcano R package was used to create volcano plots for the three pairs mentioned in the limma analysis. Second, disparities of different subgroups were shown in UpSet plots through the UpSetR R package, and the expression levels of selected DERNAs were revealed by circular heatmap by constructing the circlize R package.

### 2.4 Bioinformatics for DEmRNAs

***Functional enrichment and pathway analysis:*** Using the ClusterProfiler package and choosing an adjusted P value < 0.05 as the cut-off value and molecular function (MF) as the annotation, we performed GO analysis to DEmRNAs selected as two groups: upregulated in DMSO vs. LPS while downregulated in both LPS+AdipoAI and DMSO vs. AdipoAI; and downregulated in DMSO vs. LPS while upregulated in both LPS+AdipoAI and DMSO vs. AdipoAI. Meanwhile, KEGG analysis was performed on the DEmRNAs, and the cut-off value was set as an adjusted P value < 0.5. Finally, the results are shown in bubble diagrams created by the clusterprofile R package, and a chord diagram was plotted to show mRNAs in each pathway using the circlize R package at the same time.

***Gene set enrichment analysis:*** Gene set enrichment analysis (GSEA) is a type of analytical method for interpreting gene expression data and revealing biological pathways in common among microarray datasets (26). Using c2.cp.reactome.v7.5.1. symbols and c2.cp.kegg.v7.5.1.symbols gene sets as the background and GSEA_4.2.1 software as a tool, GSEA was performed on DEmRNAs that were divided into 3 pairs: DMSO vs. LPS; DMSO vs. AdipoAI; and LPS vs. LPS+AdipoAI.

***Construction of the Protein‒Protein Interaction (PPI) Network:*** To explore the interactions of mRNAs and inner biological mechanisms, a PPI network including 190 selected DEmRNAs was constructed by using the string database (https://cn.string-db.org/) as the background and confidence > 0.7 as the cut-off value.

### 2.5 Coexpression analysis for DEmRNAs-DElncRNAs and construction of the ceRNA network

First, to reveal the interactions between DElncRNAs and DEmRNAs, coexpression analysis was performed by the psych R package. Relevance > 0.97 and p value < 0.001 were set as the thresholds, and the ggcorrplot R package was used for visualization. Second, Cytoscape 3.9.0 was used to build the coexpression network, which was screened by Cytoscape 3.9.0 for hub genes. Since hub genes are critical in regulating networks, we chose the network with the maximum number of hubs for enrichment analyses. Thus, Gluego and GO/KEGG analyses were performed by Cytoscape 3.9.0 and Metascape (https://metascape.org/gp/index.html#/main/step1), respectively. Third, DEmRNAs and DElncRNAs in each enriched pathway were summarized for miRNA-bound DEmRNAs or DElncRNAs. TargetScan 7.2 (https://www.targetscan.org/mmu_72/) was used to predict miRNA-bound DElncRNAs, and MirWalk (http://mirwalk.umm.uni-heidelberg.de/search_genes/) was used to predict miRNA-bound DEmRNAs. Fourth, the identified coexpressed competing triplets were used to build a DEmRNA-miRNA-DElncRNA network, which was visualized by Cytoscape 3.9.0.

### 2.6 Construction of m6A-related DElncRNA networks based on coexpression analysis

To explore the relationship between m6A methylation and AdipoAI functions, m6A-related DElncRNA networks were established. First, according to previous publications (27–29), a total of 33 m6A mRNA methylation regulators (ALKBH3, ALKBH5, CBLL1, CPSF6, FMR1NB, FTO, HNRNPA2B1, IGF2BP1, IGF2BP3, IGFBP3, LRPPRC, METTL14, METTL16, METTL3, NUDT21, NXF1, PCIF1, PRRC2A, RBM15, RBM15B, RBMX, SRSF10, SRSF3, TRMT112, WTAP, XRN1, YTHDC1, YTHDC2, YTHDF1, YTHDF2, YTHDF3, ZC3H13, and ZCCHC4) were obtained and used for coexpression analysis with 110 DElncRNAs. We set the threshold as p value < 0.01 and relevance > 0.95, used the psych R package as a tool and created heatmaps by the ggcorrplot package for visualization. DElncRNAs and m6A regulator mRNAs that had interaction relationships were screened out for subsequent coexpression analysis with DEmRNAs based on Spearman analysis. Third, the m6A-related DElncRNA network was built based on DEmRNA-DElncRNA and DEmRNA-m6A regulator mRNA two-tuples by Cytoscape 3.9.0. Radar plots were used to display the expression of the m6A regulator FTO by the ggradar and ggplot2 R packages. Finally, all mRNAs from the network were summarized for GO/KEGG analysis by Metascape.

### 2.7 Validation

We selected 5 lncRNAs from the m6A-related network based on connectivity and 5 lncRNAs from 8 GO items enriched in ceRNA networks. First, a boxplot was used to reveal the expression levels of selected lncRNAs from four subgroups by the pheatmap and ggplot2 R packages. Then, the vegan package was used for the mantle order test, which is detailed in the following processes.

Total cellular RNA was extracted using a Quick-RNA Miniprep Kit (ZYMO Research, Irvine, CA, USA), and total RNA from mouse tissues was prepared with TRIzol (Life Technologies) according to the manufacturer’s instructions followed by reverse transcription and real-time quantitative polymerase chain reaction (qRT‒PCR) assays, as we described previously (L. Zhang et al., 2014). One microgram of total RNA was used for reverse transcription using M-MLV Reverse Transcriptase (Thermo Scientific, Waltham, MA, USA) according to the manufacturer’s protocol and was detected using PowerUp SYBR Green Master Mix (Thermo Scientific) on a Bio-Rad iQ5 thermal cycler (Bio-Rad Laboratories, Hercules, CA, USA). Differences in expression were evaluated by the comparative cycle threshold method using GAPDH or β-actin as a control. The primer sequences chosen for the qRT‒PCR experiments are listed in **Table 2**. Finally, Prism 8.0.2 was used to reveal the selected lncRNA expression levels by bar chart.

**Table 2:** Primer sequences chosen for the qRT‒PCR experiments.

lncRNA		Sequence (5’->3’)
	Forward primer	GGCAGCACAGGAATTTGCAG
**AK085671**	Reverse primer	TAGACACCAGAGGTTCGCCA
	Forward primer	ATAGACGCAGACCCGATTGT
**GM20632**	Reverse primer	AAATGAAGCCACCGAGCAC
	Forward primer	AGCTGCTGCTGGCTTCTTAT
**AK138558**	Reverse primer	TCTGAAACTGCTGTGAGCGA
	Forward primer	GGAGCAGCATGACCTGATGT
**PEG13**	Reverse primer	TGAGGCACCCAAGTGGAATC
	Forward primer	GCACTTCAGCGTCAAGAAGTC
**AK054221**	Reverse primer	CCCTCTCCAGTCTAGGTGTT
	Forward primer	GGCCCTGAATGGAGTAACCT
**LSS**	Reverse primer	ACCCAAGCATGAGCTACAGAA
	Forward primer	TTTCGTGGTAGCTCCTTGGTC
**NUPR1**	Reverse primer	GTGTGAGGTTAGGACAGGCA
	Forward primer	CCCGAAGCGACATTAACTGC
**CTSC**	Reverse primer	AGAGCCTCTCAACAGACGAT
	Forward primer	TGGACTGTGGGGAGTGATAGTA
**MS4A6B**	Reverse primer	ACAATTCTGGCAGGTGTGAATG
	Forward primer	ACCATGTCTGGTTGTGCAGC
**PSMB8**	Reverse primer	GACATAGGCCACCCACCTT
	Forward primer	AGGTCGGTGTGAACGGATTTG
**GADPH**	Reverse primer	TGTAGACCATGTAGTTGAGGTCA

### 2.8 Statistical analysis

The qRT-PCR data were processed with GraphPad Prism software version 8.0.1 (San Diego, CA, USA) and the mean ± standard deviation (mean ± SD) is presented for the quantitative data. Data involving more than two groups were assessed by one-way analysis of variance (ANOVA) and the level of significance was set at P-value < 0.05. The statistical analyses of microarray were performed using R software (Version 4.1.2, httpswww.r-project.org/).

## 3 Results

### 3.1 LncRNA microarray

**Figure 1:**
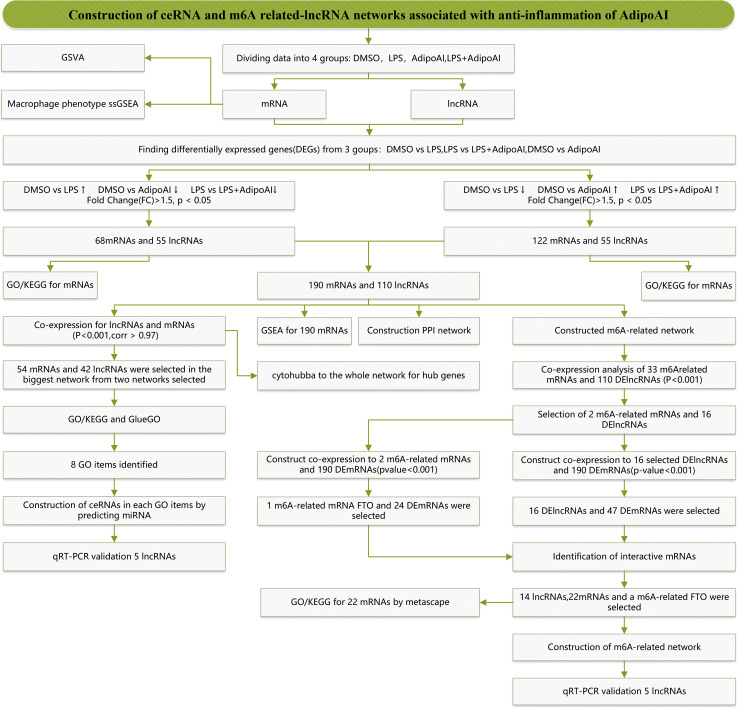
Study flow chart.

**Figure 1** displays a schematic of the workflow of the current study. Total RNA of cells in 12 samples was extracted for microarray analysis and divided into four groups. After normalization, we obtained 16553 lncRNAs and 13342 mRNAs. Box plots and principal component analysis (PCA) results displayed excellent data quality in **Supplementary Figure 1**.

### 3.2 GSVA analysis and macrophage phenotype ssGSEA

**Figure 2:**
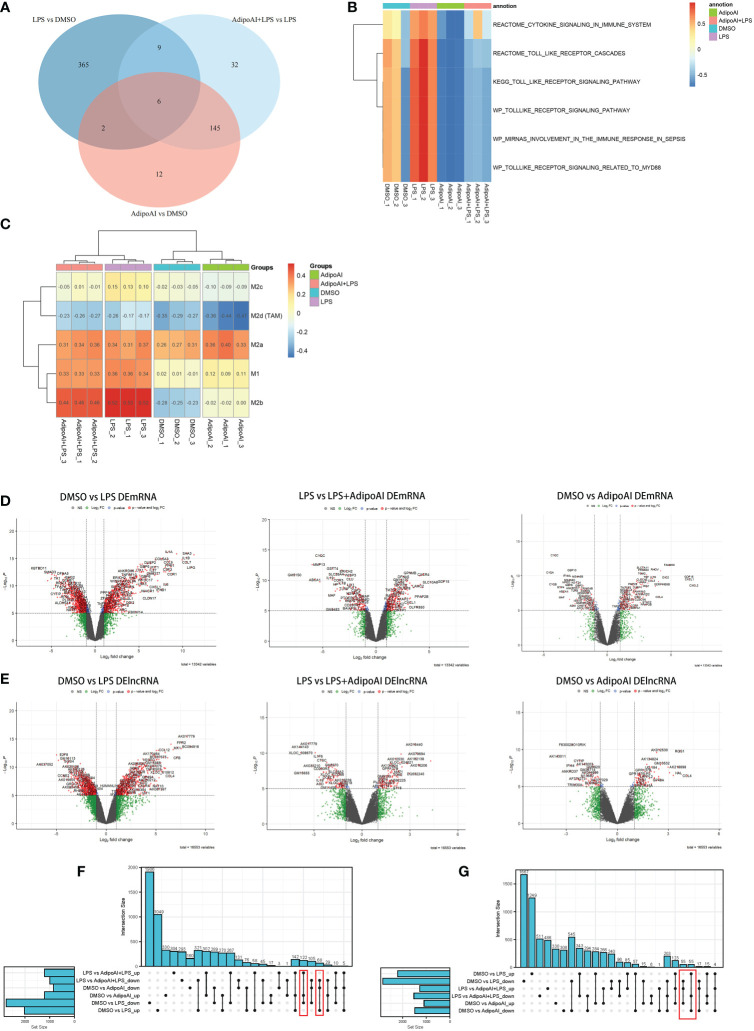
GSVA, macrophage phenotype ssGSEA and differential analysis. **(A)** Venn plot of pathways between three groups: DMSO vs. LPS, DMSO vs. AdipoAI and LPS vs. AdipoAI+LPS in GSVA. **(B)** Heatmap of GSVA scores in 12 samples. **(C)** Heatmap of macrophage phenotype ssGSEA scores in 12 samples. **(D)** Volcano plot for the mRNA differential analysis results. **(E)** Volcano plot for the lncRNA differential analysis results. **(F, G)** UpSet plots for the differentially expressed genes of **(F)** DEmRNAs or **(G)** DElncRNAs.

GSVA was performed on mRNAs from 12 samples, and 928 related pathways were enriched, while a heatmap revealed the scores in **Supplementary Figure 2**. The limma R package was used to identify differentially expressed pathways in the three groups, and the results are shown in **Supplementary Figure 3**. After that, intersection pathways among these groups were selected and are shown in **Figure 2A**. Six pathways were identified as the intersection of the three groups, and their GSVA scores are shown in **Figure 2B**. Furthermore, to explore the changes in macrophage phenotypes in each group, ssGSEA was conducted and found that AdipoAI could effectively reduce the LPS-induced phenotypic changes of macrophages from the M2b type to the M2c type (**Figure 2C**).

### 3.3 Identification of 190 DEmRNAs and 110 DElncRNAs

**Figure 3:**
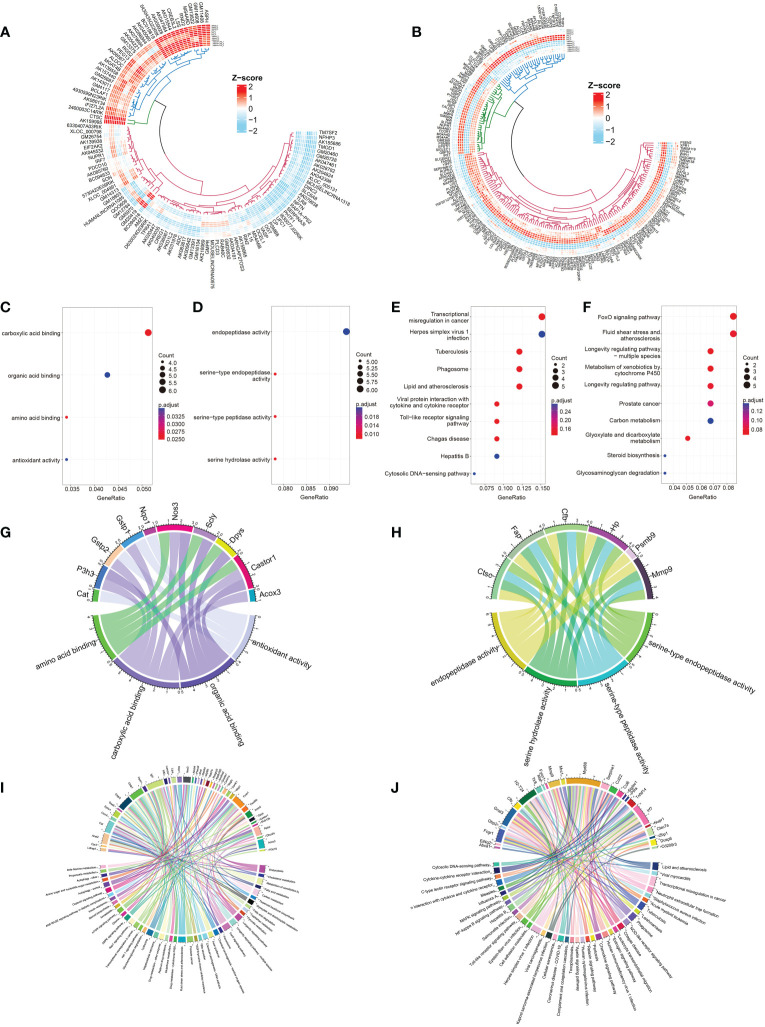
Identification and selection of 190 DEmRNAs/110 DElncRNAs and enrichment analysis of the two groups of DEmRNAs. **(A, B)** Circular heatmaps show the common differentially expressed genes among the three groups (LPS vs. DMSO, AdipoAI vs. DMSO and AdipoAI+LPS vs. LPS). **(A)** DElncRNAs. **(B)** DEmRNAs. **(C)** Molecular function enrichment analysis of 122 DEmRNAs (DMSO vs. LPS↓, DMSO vs. AdipoAI↑ and LPS vs. LPS+AdipoAI↑). **(D)** KEGG for 122 DEmRNAs. **(E)** Molecular function enrichment analysis of 68 DEmRNAs (DMSO vs. LPS↑, DMSO vs. AdipoAI↓ and LPS vs. LPS+AdipoAI↓). **(F)**: KEGG for 68 DEmRNAs. **(G–J)** Bubble diagrams showing mRNAs involved in molecular function enrichment analysis and KEGG. **(G)** 122 DEmRNAs in molecular function enrichment analysis. **(H)** 122 DEmRNAs in KEGG. **(I)** 68 DEmRNAs in molecular function enrichment analysis. **(J)** 68 DEmRNAs in KEGG.

To discover the molecular mechanism of the AdipoAI-related anti-inflammatory effect, the limma package was used to identify DEmRNAs and DElncRNAs related to AdipoAI. A volcano plot **(** **Figures 2D, E** **)** was used to show the results between the 3 subgroups. Upset plots created by UpSetR R package are shown in **Figures 2F, G** to describe the intersecting lncRNAs and mRNAs between different groups. Fifty-five DElncRNAs and 68 DEmRNAs were identified as the first group that were upregulated in DMSO vs. LPS but downregulated in both LPS vs. LPS+AdipoAI and DMSO vs. AdipoAI. Simultaneously, 55 DElncRNAs and 122 DEmRNAs were identified as the second group, which was downregulated in DMSO vs. LPS but upregulated in both LPS vs. LPS+AdipoAI and DMSO vs. AdipoAI. Ultimately, 110 DElncRNAs and 190 DEmRNAs were identified and selected for subsequent processes. In addition, a circular heatmap created by the circlize R package **(** **Figures 3A, B** **)** was used to show the expression levels of 110 DElncRNAs and 190 DEmRNAs in 12 samples.

### 3.4 Bioinformatics analysis of 190 DEmRNAs

***Enrichment analysis of DEmRNAs:*** Bubble diagrams **(** **Figures 3C–F** **)** revealed the results of GO/KEGG analysis of DEmRNAs in each selected group. A p value < 0.5 was set as the threshold of KEGG, and a p value < 0.05 was set as the threshold for GO analysis. A total of 4 GO terms and top 10 KEGG pathways were enriched in the first group, while 4 GO terms and top 10 KEGG pathways were enriched in the second group. A chord diagram was plotted to show the mRNAs in each pathway **(** **Figures 3G–J** **)**.

**Figure 4:**
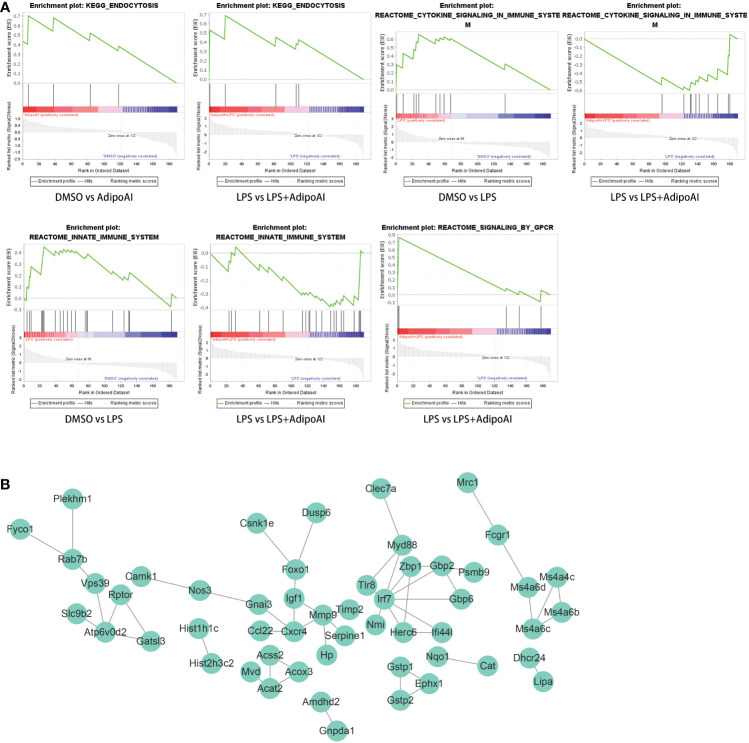
GSEA and PPI network for 190 DEmRNAs. **(A)** Gene set enrichment analysis of 190 DEmRNAs. **(B)** PPI network with 53 nodes and 53 edges of 190 DEmRNAs.

***GSEA for the selection of potential AdipoAI-related pathways:*** The function of AdipoAI was inferred from the functions of DEmRNAs selected previously; thus, GSEA was performed on 190 DEmRNAs. Against the background of the KEGG database, both LPS vs. LPS+AdipoAI and DMSO vs. AdipoAI enriched a similar pathway when AdipoAI was added: ENDOCYTOSIS. In addition, based on the Reactome database, 2 similar pathways were enriched upon addition of LPS alone in both DMSO vs. LPS and LPS vs. LPS+AdipoAI: REACTOME_SIGNALING_BY_GPCR. Moreover, **Figure 4A** displays enrichment plots for each enriched pathway screened out by GSEA.

***Construction of the PPI network:*** **Figure 4B** shows the PPI network built by the STRING online database with 53 nodes and 53 edges. Diagrams were downloaded from the STRING website.

### 3.5 Construction of the ceRNA network with hub codes

**Figure 5:**
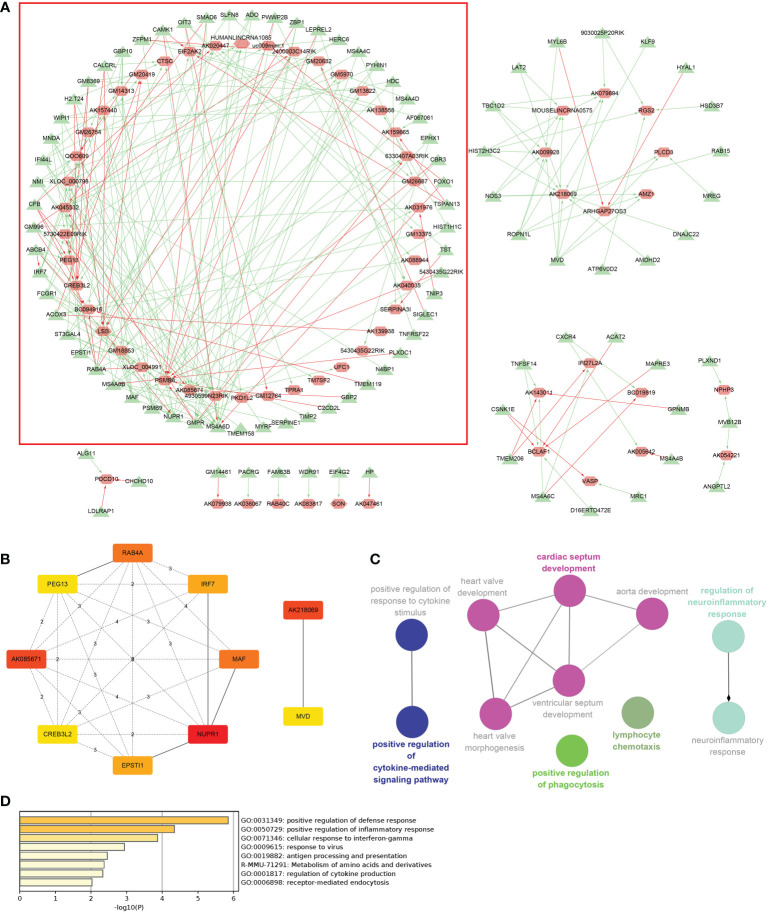
Coexpression analysis for DElncRNAs-DEmRNAs. **(A)** The coexpression network of DElncRNAs-DEmRNAs and the largest network with the most hub nodes in the red frame were selected (lncRNA: red; mRNA: green). **(B)** Hub nodes in all networks. **(C)** Gluego for 58 mRNAs in the largest network. **(D)** GO analysis for 58 mRNAs in the largest network.

**Figure 6:**
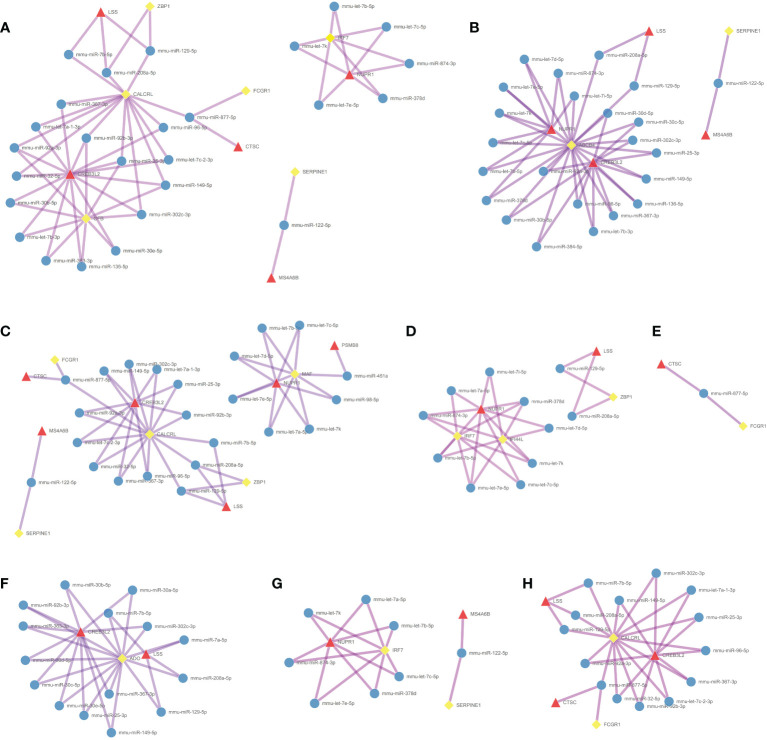
Construction of the ceRNA networks. DEmRNA-miRNA-DElncRNA networks of **(A)** positive regulation of defense response, **(B)** cellular response to interferon-gamma, **(C)** positive regulation of inflammatory response, **(D)** response to virus, **(E)** antigen processing and presentation, **(F)** metabolism of amino acids and derivatives, **(G)** regulation of cytokine production, and **(H)** receptor-mediated endocytosis. (Blue Ball: miRNA; Red Triangle: lncRNA; Yellow Square: mRNA).

Coexpression results between DElncRNAs and DEmRNAs were visualized by heatmap **(** **Supplementary Figure 4** **)**. **Figure 5A** shows the coexpression network built by 190 DEmRNAs and 110 DElncRNAs with relevance > 0.97 and P value < 0.001. The cytoHubba results are shown in **Figure 5B**. We chose the network with the most hub nodes, which contained 58 mRNAs and 42 lncRNAs (**Figure 5A** with Red Frame). Fifty-eight mRNAs were screened by Gluego analysis and are displayed in **Figure 5C**. After that, GO/KEGG analysis was performed using Metascape, and 8 GO terms were identified, as shown in **Figure 5D**. MiRwalk and TargetScan were used to reverse-predict miRNA-bound DEmRNAs and miRNA-bound DElncRNAs, respectively. Therefore, based on the DEmRNA-miRNA-DElncRNA axis in each GO term, ceRNA networks for each term were separately built by Cytoscape and are shown in **Figures 6A–H**.

### 3.6 Identification of m6A-related DElncRNAs and construction of an m6A-related DElncRNA network

**Figure 7:**
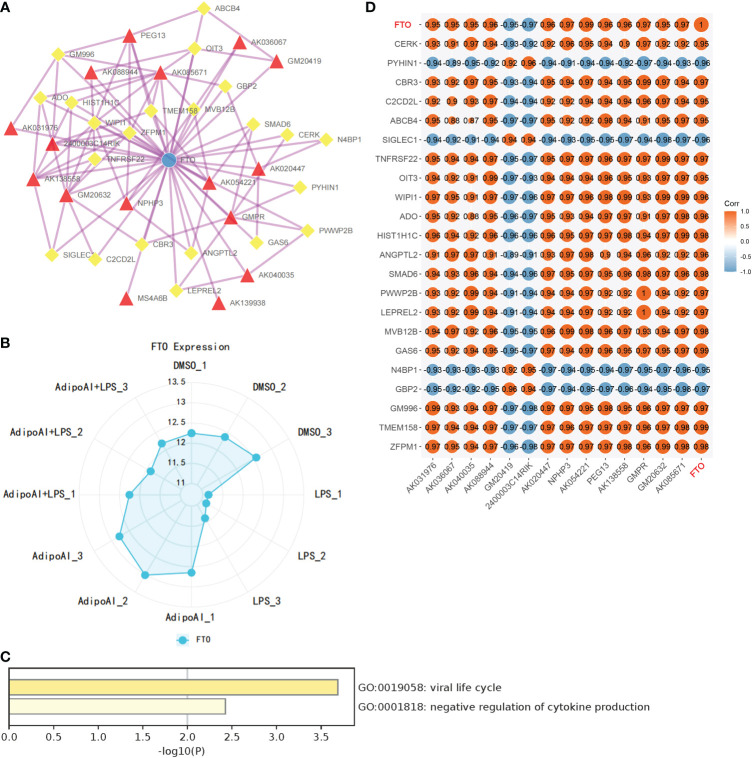
Construction of m6A regulator lncRNA‒mRNA networks. **(A)** FTO (M6A regulator)-lncRNA‒mRNA network. (Blue Ball: m6A regulator; Red Triangle: lncRNA; Yellow Square: mRNA). **(B)** FTO expression levels in 12 samples. **(C)** GO analysis for mRNAs in the network. **(D)** Coexpression validation for FTO, mRNAs and lncRNAs in networks.

Thirty-three m6A-related mRNAs were retrieved from a previous study, used to perform a coexpression analysis (Spearman) with 110 previously selected DElncRNAs and visualized by heatmap (**Supplementary Figure 5**). Then, two m6A-related RNAs and 16 DElncRNAs were screened out, and a heatmap is shown in **Supplementary Figure 6**. To further confirm the relationship between m6A methylation and AdipoAI-related DERNAs, Spearman analysis of coexpression was performed between 190 DEmRNAs with 2 m6A-related RNAs and 16 DElncRNAs selected previously. Then, the m6A-related mRNA FTO with 24 associated DEmRNAs and 16 DElncRNAs with 47 associated DEmRNAs was screened out (**Supplementary Figures 7**, **8**). Afterwards, we selected the intersection of these results and obtained 14 DElncRNAs, 22 DEmRNAs and the m6A-related mRNA FTO. Based on their inner interactions, the m6A-related DElncRNA network was established and visualized by Cytoscape (**Figure 7A**). Furthermore, the expression level of the central mRNA FTO was detected, and the radiogram is displayed in **Figure 7B**. GO analysis was performed on 22 DEmRNAs and resulted in two identified GO terms, as shown in **Figure 7C**. Finally, a coexpression heatmap was created to verify the results based on 14 DElncRNAs, 22 DEmRNAs and FTO, as shown in **Figure 7D**.

### 3.7 qRT‒PCR validation of 10 DElncRNAs

**Figure 8:**
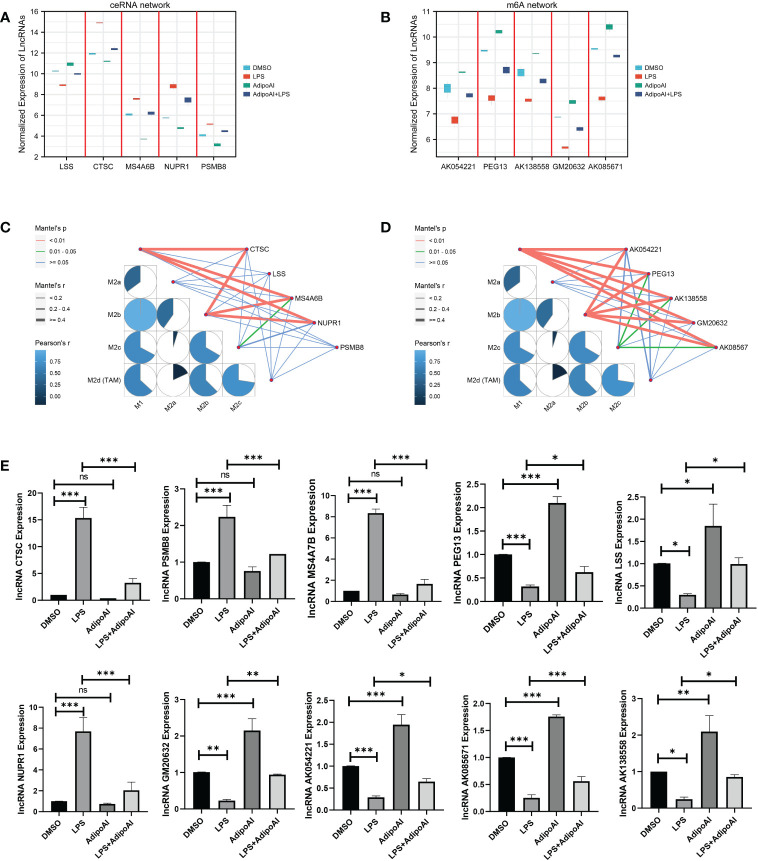
Microarray and qRT‒PCR validation. **(A)** Box plot for the expression of 5 lncRNAs selected in ceRNA networks. **(B)** Box plot for the expression of 5 lncRNAs selected in the m6A-regulated lncRNA‒mRNA networks. **(C, D)** Mantle order test of lncRNAs selected from the ceRNA- and m6A-related DElncRNA networks. **(C)** ceRNA networks, **(D)** m6A-related DElncRNA networks. **(E)** qRT‒PCR validation for 10 lncRNAs (n=3). ns: no significant, * P <0.05, ** P <0.01, *** P <0.001.

To further confirm the functions of the 10 AdipoAI-regulated DElncRNAs selected from the ceRNA and m6A-related DElncRNA networks, a box plot was created to reveal their expression levels among 12 samples, as shown in **Figures 8A, B**. The results of the mantle order test suggested that most lncRNAs in the ceRNA and m6A networks were related to M2b and M1 macrophage phenotypes (**Figures 8C, D**). qRT‒PCR was used to verify the results shown in **Figure 8E**. Both data validation and qRT‒PCR results showed that these selected DElncRNAs were regulated by AdipoAI.

## 4 Discussion

Although emerging evidence has shown that APN and its receptor agonists feature anti-inflammatory effects (7, 13), the precise molecular mechanisms remain unclear, especially the functions of lncRNAs. Thus, the current research aimed to provide mechanistic insight into the role played by lncRNAs in the AdipoAI-related anti-inflammatory effect, which attenuated LPS-induced inflammation in macrophages. More importantly, m6A methylation and ceRNA interactions were set as the entry points for further discovering how lncRNAs work in anti-inflammatory effects and macrophage polarization. Transcriptome sequencing and a series of bioinformatic analyses were conducted to elucidate these mechanisms at the high-throughput level.

Initially, enrichment analysis was conducted through GSVA, and the results indicated that AdipoAI inhibited the signal transduction of cytokines in the immune system and the activation of Toll-like receptor signaling pathways, especially signals associated with MYD88. LPS has been proven to affect inflammatory progression and the immune response by inducing cells through TLR4. Notably, TLR4-triggered signal transduction relies on the adaptor proteins myeloid differentiation marker 88 (MyD88) and adaptor-inducing IFNβ (TRIF), which mediate MyD88- and TRIF-dependent signaling pathways while simultaneously containing the Toll-interleukin-1 (IL-1) receptor (TIR) domain (30, 31). According to the enrichment analysis, AdipoR1/APPL1 stably interacted with MyD88. This complex inhibited the activation of the NF-kB, MAPK, and c-Maf pathways and restricted the LPS-induced production of proinflammatory cytokines in macrophages. A previous study was found and was consistent with our results, which strongly supported the reliability of our findings (7).

Differentially expressed lncRNAs and mRNAs were identified among 12 samples, and 110 DElncRNAs and 190 DEmRNAs were screened out and divided into two groups. Since the mechanism of AdipoAI-related anti-inflammatory effects relies on the proteins and RNAs affected by AdipoAI, GO/KEGG analysis was performed for pathway enrichment. Then, GO terms of antioxidant activity, serine and endopeptidase and KEGG pathways, including FoxO signaling and Toll-like receptor signaling, were identified. Notably, antioxidants play essential roles in the regulation of the immune response and inflammation restriction (32), and serine is necessary for LPS-induced expression of IL-1β in macrophages (33). Additionally, the FoxO signaling pathway also regulates innate immune cells (34). Hence, with various enriched pathways closely interacting with the immune response, AdipoAI was proven to be involved in anti-inflammatory mechanisms, particularly in macrophages.

To further clarify the function of AdipoAI on enriched pathways, GSEA was performed on 190 DEmRNAs. Surprisingly, pathways related to cytokines and the innate immune response were inhibited, while the endocytosis pathway was activated. It has been suggested that the extracellular domain of TLR4 is indispensable for LPS-induced endocytosis (35), and TLR4-related endocytosis is necessary for the signal generation, receptor degradation and signal termination of TRIF-dependent proinflammatory factors (36, 37). Affecting the endocytosis of AdipoR1 may stimulate the phosphorylation of AMPK and ACC, which is mediated by APN and hormones (38, 39). Consequently, endocytosis may contribute to the signaling regulation of APN. In addition, GSEA and other results showed that AdipoAI had a significant correlation with endocytosis; that is, it might serve as the core pathway of AdipoAI-related anti-inflammatory effects.

Recently, a significant moderating role of large-scale biomechanisms has necessitated the investigation of ncRNAs in different fields. lncRNAs and miRNAs are the main constituents of ncRNAs and participate in various biological processes, such as immune responses, by moderating immune-related genes (40, 41). Among them, lncRNAs were identified as transcripts more than 200 nucleotides in length without protein-coding potential (41, 42). In 2011, the ceRNA hypothesis was proposed based on coexpressed competing triplets built by lncRNAs, miRNAs and mRNAs. This suggests a novel theory that lncRNAs and mRNAs compete for binding with one interacting miRNA, thus regulating each other (43) and affecting the miRNA-related negative regulation of gene expression. In previous investigations related to the ceRNA theory and anti-inflammatory effects, lncRNAs have been shown to play an essential role. For example, lncRNAs have been reported to regulate p38 mitogen-activated protein kinase and the nuclear factor-kb signaling pathway through the linc00707/mir-223-5p axis in LPS-induced mrc-5 cells (44). Additionally, lncRNA SNHG16 can upregulate TLR4 through and moderate the miR-15a/16 cluster through the ceRNA network to affect LPS-induced inflammatory pathways (45).

Making use of this theory, we constructed a ceRNA network to further study the mechanistic role played by lncRNAs in AdipoAI-regulated anti-inflammation. Then, a coexpression network was built, and the largest cluster was selected for enrichment analysis, in which 8 terms were identified: positive regulation of defense response, positive regulation of inflammatory response, cellular response to interferon-γ, antigen processing and presentation, regulation of cytokine production, metabolism of amino acids and derivatives and receptor-mediated endocytosis. Among them, a group of items, including regulation of cytokine production, antigen processing and presentation, cellular response to interferon-γ, and receptor-mediated endocytosis, were speculated to be the potential key pathways of AdipoAI-related anti-inflammatory effects since they were reported to be associated with the immune response of macrophages. Given that interferon-γ can activate macrophages and their sensitivities to TLR-induced cellular death (46), these pathways may contribute to the molecular mechanism of AdipoAI-related anti-inflammatory effects. Using TargetScan and mirWalk, we predicted and formed lncRNA‒miRNA-mRNA triplets and built ceRNA networks based on triplets in each enriched GO item from the coexpression analysis. Finally, networks were constructed and provided new insight into the mechanistic theory for the role played by lncRNAs in AdipoAI-related anti-inflammatory effects based on the ceRNA theory. Furthermore, 5 lncRNAs from networks were selected as potential therapeutic targets for inflammation since they were discovered to participate in the anti-inflammatory effect caused by APN receptor agonists.

As noted by a recent publication (47), which studied the association between m6A methylation and LPS-induced inflammation in macrophages, the key enzyme for m6A methylation, METTL3, is closely associated with the immune response and inflammatory regulation. Moreover, the expression and biological activity of METTL3 could be enhanced by LPS, while overexpression may significantly reduce the severity of LPS-induced inflammation in macrophages. METTL3 influences these processes through the NF-kB pathway (47). Accordingly, we hypothesized that m6A methylation may contribute to the function of lncRNAs in AdipoAI-related anti-inflammatory effects. Twenty-three mRNAs related to methylation were obtained (27, 28) from recent publications and used for coexpression analyses. A network with the central mRNA FTO was built based on the coexpression relationships among DElncRNAs, DEmRNAs and m6A regulators, revealing the interaction between AdipoAI and m6A methylation. FTO is reported to positively correlate with the expression of APN (48), and the reduction in FTO can inhibit the NLRP3 inflammasome through the FoxO1/NF-kB signaling pathway in macrophages (49). Hence, AdipoAI may regulate the m6A methylation regulator FTO to influence related RNAs, thus affecting immune-related pathways and resulting in an anti-inflammatory effect. Obviously, FTO provides a theoretical basis for the interaction between m6A methylation and anti-inflammatory effects.

Furthermore, as demonstrated by enrichment analysis of mRNAs in m6A networks, these m6A-related mRNAs can suppress the secretion of cytokines. Thus, we speculated that AdipoAI might regulate the secretion of cytokines through the coexpression network. Among the network, lncRNA Peg13 regulates the Wnt/β-catenin pathway through the mir-490-3p/psmd11 axis (50) and attenuates the toxicity of sevoflurane to neural stem cells through the absorption of mir-128-3p while protecting the expression of SOX13 (51). At the same time, Peg13 can also alleviate hypoxic-ischaemic brain injury in neonatal mice *via* the mir-20a-5p/XIAP axis (52). In other studies, knockdown of the m6A-binding protein ythdf2 increased the expression levels of map2k4 and map4k4 mRNA by stabilizing mRNA transcripts. Similarly, the YTHDF2 protein can activate the MAPK and NF-kB signaling pathways and exacerbate the inflammatory reaction in LPS-induced primary Raw264.7 cells to promote the expression of proinflammatory cytokines (53). Other research (54) proved that LPS treatment promoted Socs1 m6A methylation and sustained SOCS1 induction by promoting FTO degradation. Interestingly, their purpose was to simulate the phenotype of METTL14-deficient macrophages by forcing FTO expression in macrophages, while the results are consistent with ours, strongly proving the reliability of our analyses. As a consequence, we discovered that Socs1 inhibited signal transduction, while AdipoAI suppressed cytokine secretion by affecting related genes; thus, AdipoAI played a role in anti-inflammation by inhibiting the degradation of FTO.

To illuminate how AdipoAI works to influence macrophage phenotypes, ssGSEA was used to reveal scores of different phenotypes in each group of DEmRNAs. The results suggested that AdipoAI could reduce the LPS-induced phenotypic change from the M2b to the M2c type. A previous study (24) showed that the M2b type is associated with tumor progression, immune regulation and the Th2-related response. The M2c type is related to phagocytosis of apoptotic bodies, tissue remodeling and immune suppression (24). In accordance with these publications, AdipoAI may play a key role in macrophage phenotypic changes. The mantle order test suggested that the following genes were related to the M1 and M2b macrophage phenotypes: CTSC, MS4A6B, NUPR1, PEG13, AK054221, AK138558, GM20632, and AK08567. Thus, these genes are critical in both macrophage polarization and AdipoAI-related anti-inflammatory effects.

Notably, all of the sequencing data we extracted were from mice, which may lead to certain limitations. More specific analyses and *in vivo* experiments are necessary for further elucidation of the mechanisms of these newly identified lncRNAs.

In summary, we screened lncRNAs as candidate regulators of the anti-inflammatory mechanism of AdipoAI. Moreover, we identified specific lncRNAs from these processes based on m6A-related DElncRNAs and ceRNA networks, providing a novel reference for the subsequent exploration of the molecular mechanism of AdipoAI-related anti-inflammatory effects.

## Data availability statement

The datasets presented in this study can be found in online repositories. The names of the repository/repositories and accession number(s) can be found below: GEO (GSE212065).

## Author contributions

HY, FF, and WQ contributed to the conception and design of the research. HY, HW, QX, and WQ contributed to the writing and drafting of the manuscript. ZL and ZC contributed to drawing the figures and tables and analyzing the data. QT, JC, and FF contributed to the critical revision of the manuscript for important intellectual content. All the authors have approved the final version of the manuscript to be published and agree to be accountable for all aspects of the work.

## Supplementary material

Supplementary Figure 1 Quality control for lncRNA microarray. **(A)** PCA of lncRNA expression profiles. **(B)** PCA of mRNA expression profiles. **(C)** Boxplot of lncRNAs. **(D)** Boxplot of mRNAs.

Supplementary Figure 2 Pheatmap for 928 pathway GSVA scores.

Supplementary Figure 3 Volcano plot for differential pathways in the 4 groups. **(A)** Differential pathways: DMSO vs. AdipoAI. **(B)** Differential pathways: DMSO vs. LPS. **(C)** Differential pathways: LPS vs. AdipoAI+LPS.

Supplementary Figure 4 Coexpression between DEmRNAs and DElncRNAs.

Supplementary Figure 5 Coexpression between 33 m6A-related mRNAs and 110 DElncRNAs.

Supplementary Figure 6 Coexpression between 2 m6A-related mRNAs and 16 lncRNAs.

Supplementary Figure 7 Coexpression between 2 m6A-related mRNAs and 190 DEmRNAs.

Supplementary Figure 8 Coexpression between 16 lncRNAs and 190 DEmRNAs.
