# Supplementary figures and images for: Construction of ceRNA and m6A-related lncRNA networks associated with anti-inflammation of AdipoAI
Source: Front Immunol. 2023 Jan 10;13:1051654. doi: 10.3389/fimmu.2022.1051654 (PMC9871488; doi:10.3389/fimmu.2022.1051654)

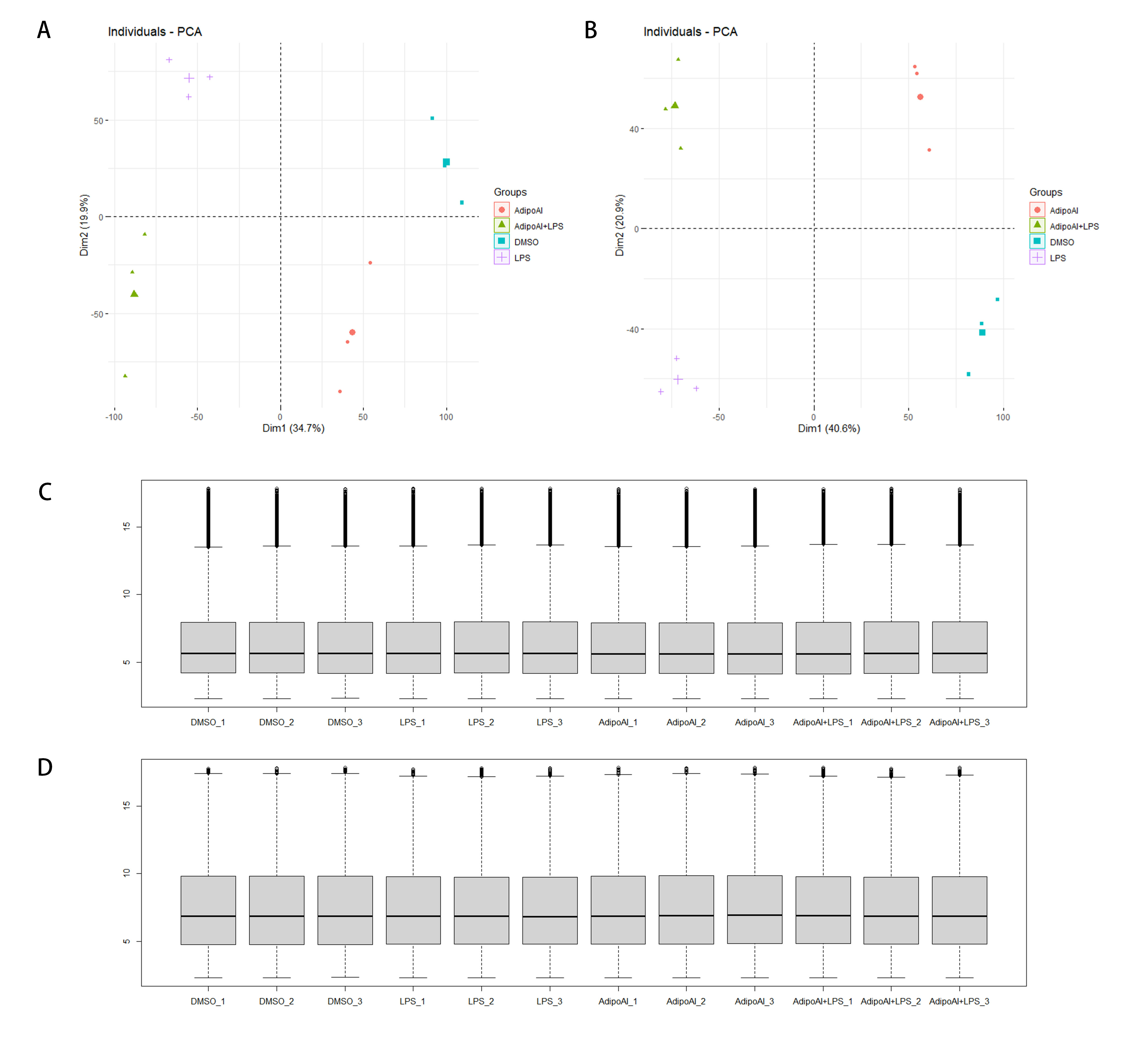

Supplement: Supplementary Figure 1 — Quality control for lncRNA microarray. (A) PCA of lncRNA expression profiles. (B) PCA of mRNA expression profiles. (C) Boxplot of lncRNAs. (D) Boxplot of mRNAs. [file Image_1.tif]

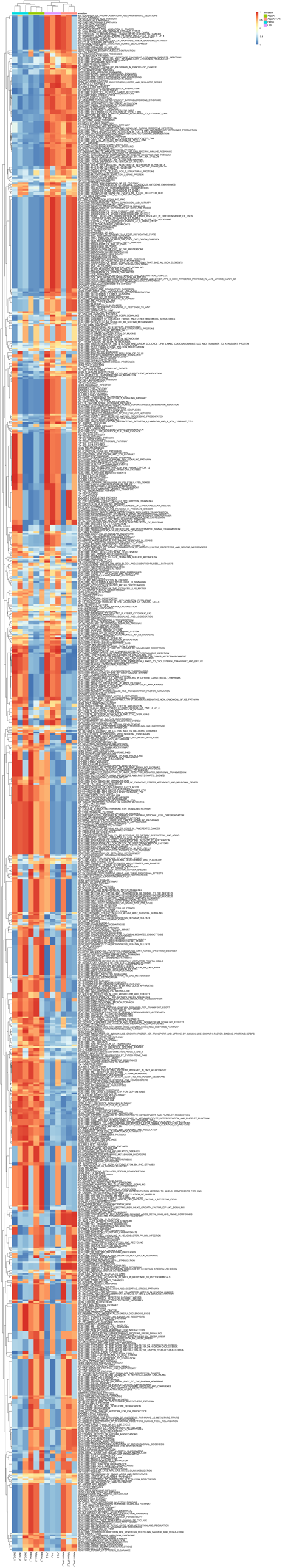

Supplement: Supplementary Figure 2 — Pheatmap for 928 pathway GSVA scores. [file Image_2.tif]

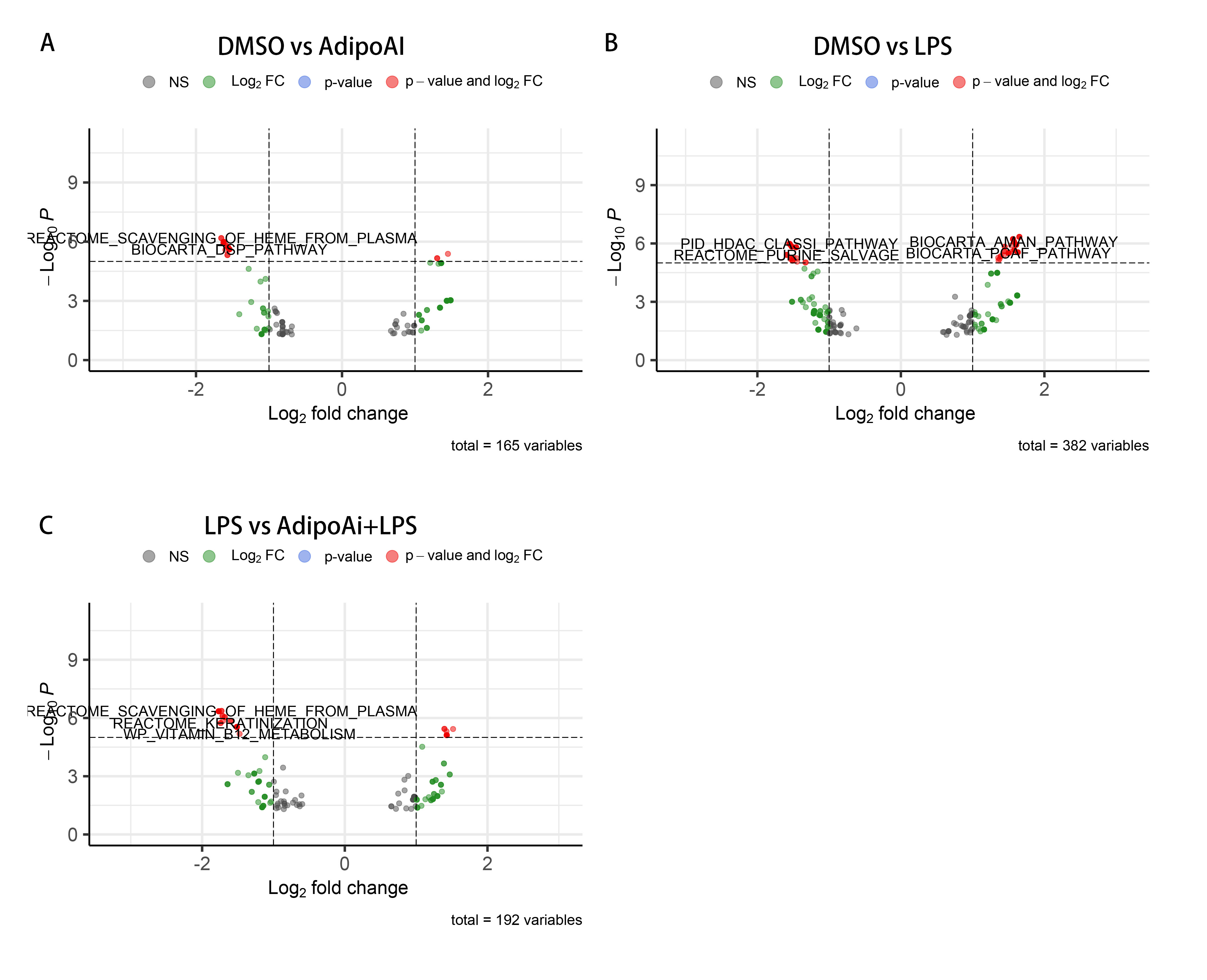

Supplement: Supplementary Figure 3 — Volcano plot for differential pathways in the 4 groups. (A) Differential pathways: DMSO vs. AdipoAI. (B) Differential pathways: DMSO vs. LPS. (C) Differential pathways: LPS vs. AdipoAI+LPS. [file Image_3.tif]

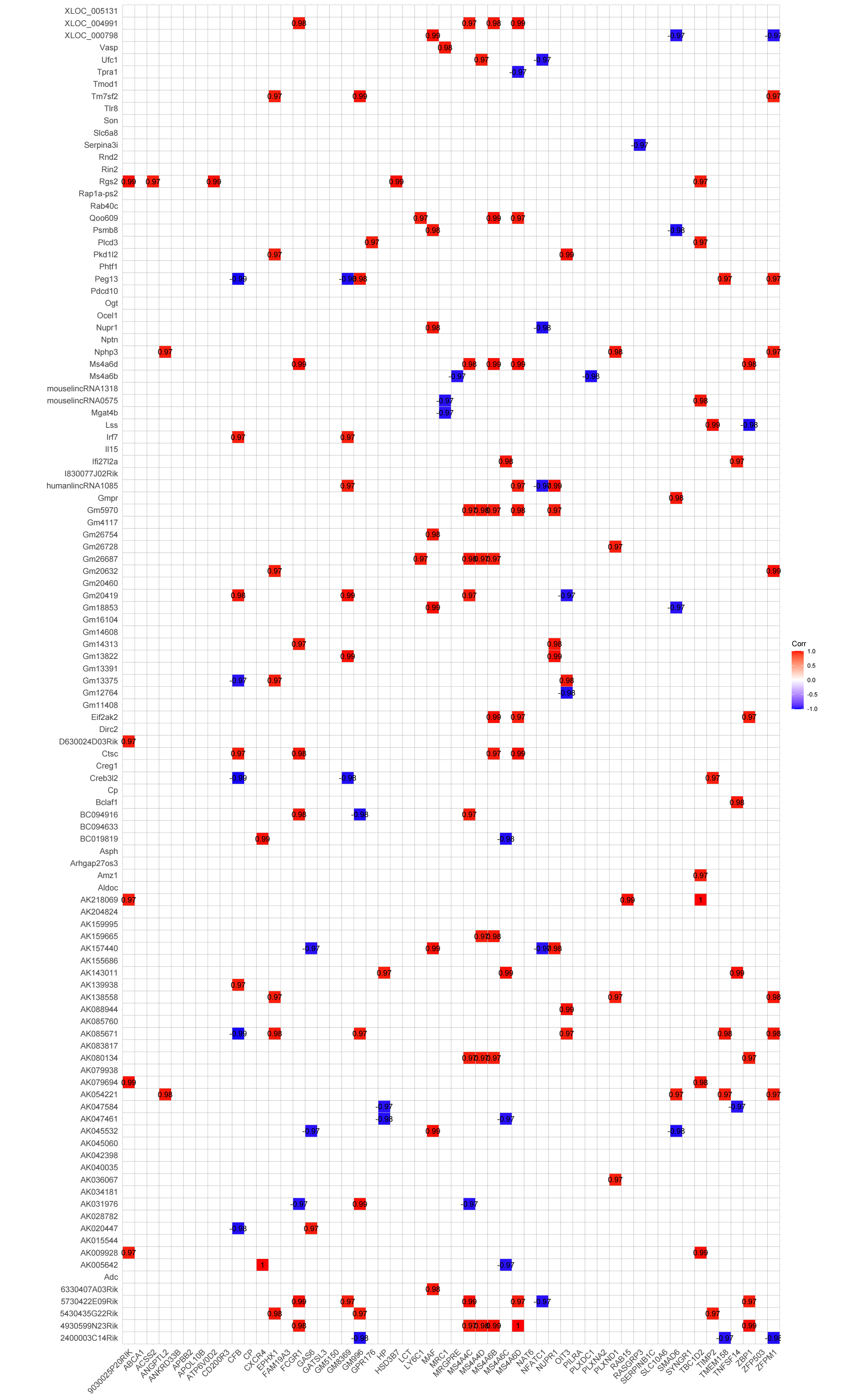

Supplement: Supplementary Figure 4 — Coexpression between DEmRNAs and DElncRNAs. [file Image_4.tif]

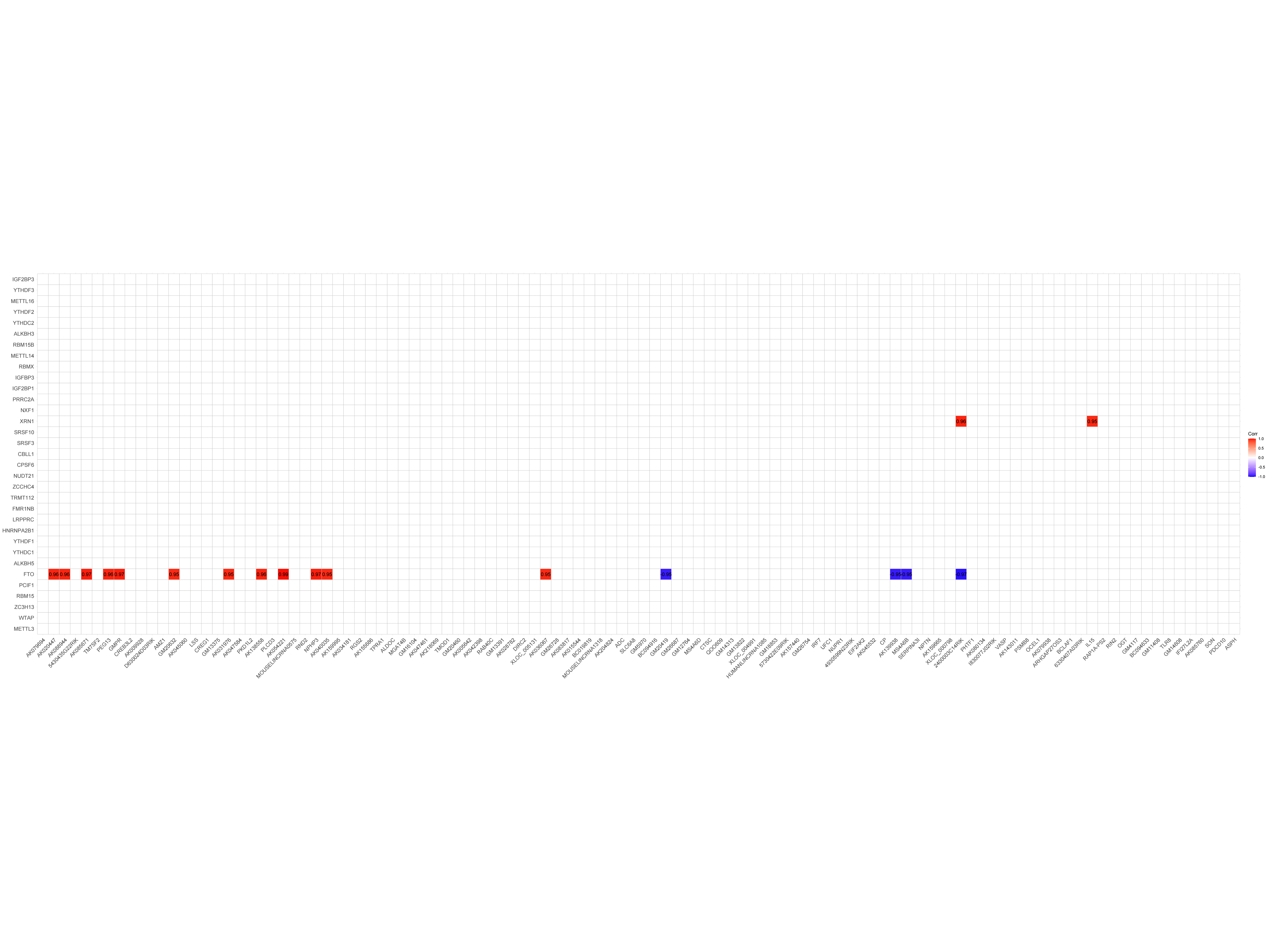

Supplement: Supplementary Figure 5 — Coexpression between 33 m6A-related mRNAs and 110 DElncRNAs. [file Image_5.tif]

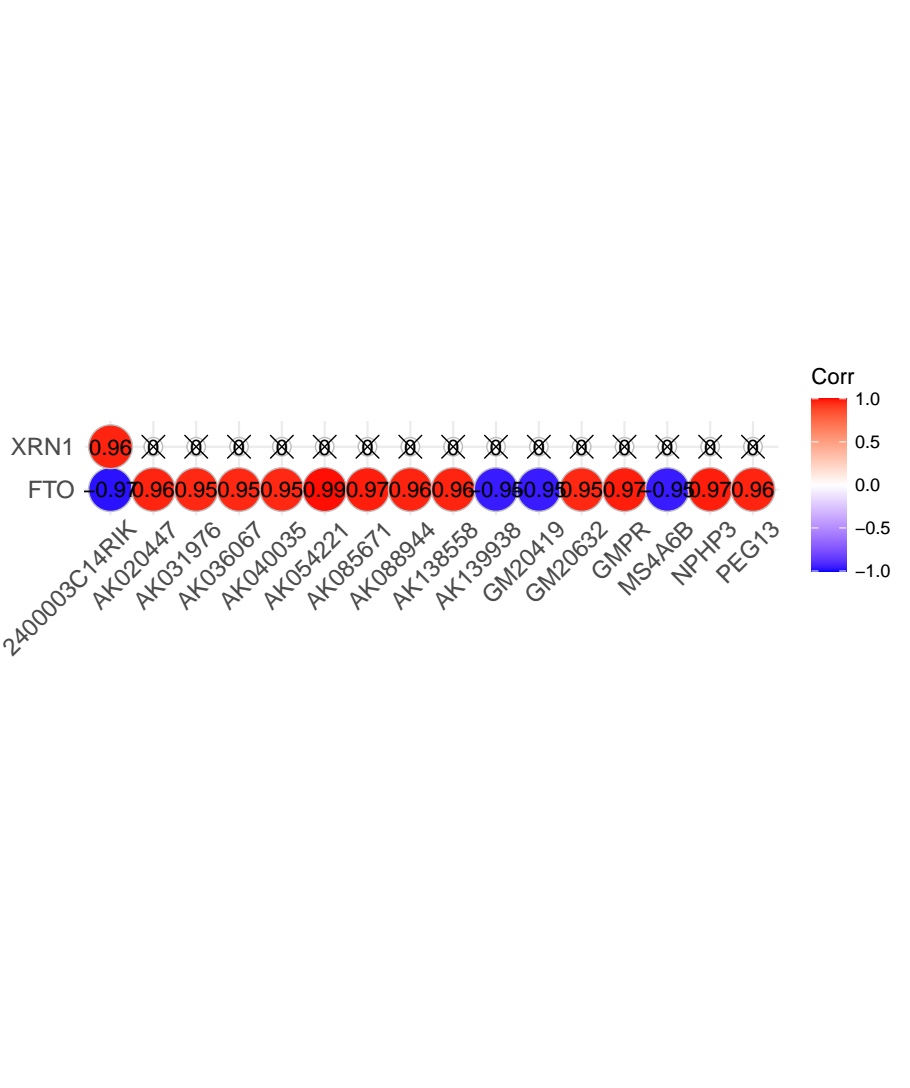

Supplement: Supplementary Figure 6 — Coexpression between 2 m6A-related mRNAs and 16 lncRNAs. [file Image_6.tif]

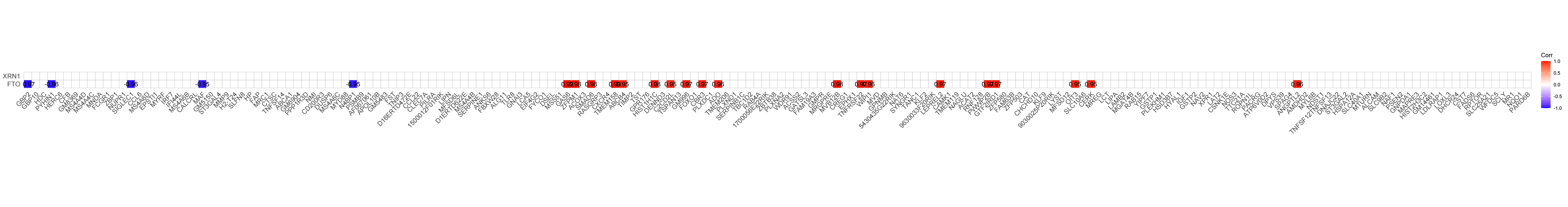

Supplement: Supplementary Figure 7 — Coexpression between 2 m6A-related mRNAs and 190 DEmRNAs. [file Image_7.tif]

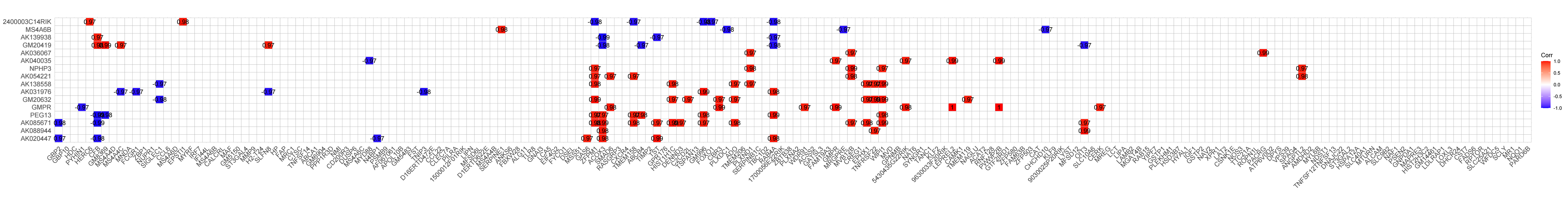

Supplement: Supplementary Figure 8 — Coexpression between 16 lncRNAs and 190 DEmRNAs. [file Image_8.tif]
